# Atypical Chest Pain in a Patient With Breast Implant

**DOI:** 10.7759/cureus.37751

**Published:** 2023-04-18

**Authors:** Khairul Anuar Azis, Muath Mamdouh Mahmod Al-Chalabi, Siti Fatimah Noor Mat Johar, Wan Azman Wan Sulaiman

**Affiliations:** 1 Reconstructive Sciences Unit, Universiti Sains Malaysia (USM), Kota Bharu, MYS

**Keywords:** silicone implant rupture, atypical chest pain, breast implant illness, breast implant complications, breast implant

## Abstract

Breast implant surgery typically improves patient breast satisfaction and health-related quality of life. However, breast implants are also linked to long-term local problems like capsular contracture and breast discomfort. Chest pain is one of the reasons that patients with breast implants seek consultations, which is not typically attributable to cardiovascular reasons. The potential reasons for atypical chest pain are diverse. The absence of a precise diagnosis may also result in incorrect examinations and management, leading to further worry and wasted work time.

A 55-year-old woman with a breast implant 10 years prior to the incident, presented with atypical chest pain on and off for a year and was treated as a case of unstable angina, costochondritis, and vasospastic spasm. Despite multiple visits, her symptoms did not resolve. Later, the patient presented with a lump over the left breast, associated with constitutional symptoms. Examination revealed a left breast implant with capsular contracture grade III, and ultrasonography showed signs of a ruptured implant. Symptoms eventually resolved after the removal of the breast implant.

## Introduction

During World War II, Japanese ladies who wanted larger breasts underwent direct injections of paraffin or silicone [1]. Since Cronin created breast implants from silicon in the early 1960s, these devices have been a crucial component of plastic surgeons' cosmetic and reconstructive toolkits worldwide [2]. Silicone gel-filled devices have seen tremendous design progress and intense worldwide scrutiny and safety debate over the decades. Safety concerns ultimately brought about a silicone implant ban in the US from 1992 to 2000. However, extensive research disproved claims that breast implants were associated with breast cancer and connective tissue disorders. Although the link between breast implants and autoimmune and rheumatic diseases is debatable, scientific evidence failed to demonstrate that breast implants caused disease, and the FDA lifted the moratorium on silicone breast implants for women over the age of 22 in November 2006, allowing their use [3].

Non-Cardiac Chest Pain (NCCP) is defined as recurring substernal chest pain of non-cardiac origin. It may be of musculoskeletal, pulmonary, gastroenterological, psychosomatic, or neurologic etiology. Although NCCP affects both sexes, middle-aged women are much more likely to be affected than males [4]. In addition, breast implants have been linked to many general side effects, including infection, leaking, and capsular contracture, according to published research. In this case report, a breast implant with an intracapsular rupture that mimicked coronary artery disease is used to demonstrate how breast implants can also result in unusual problems and symptoms in middle-aged women.

## Case presentation

A 55-year-old woman with an underlying anxiety disorder was referred to us by the cardiologist for a suspected ruptured left breast implant. She had undergone a bilateral breast augmentation with silicone implants 10 years ago. The patient had visited the emergency department recently because of chest tightness, decreased effort tolerance, and exertional dyspnea. She had been experiencing the symptoms worsening a week before the presentation. There was no known medical condition other than her anxiety issue, which does not require medication. The patient denied using standard drugs, smoking, or having a family history of either breast cancer or heart disease.

Her blood pressure was 119/65 mmHg, her pulse rate was 94 beats per minute, and her cardiovascular and respiratory systems were normal. No other obvious abnormality was noted on examination. The electrocardiogram (ECG) revealed right ventricular strain with a HEART score of five, and the patient was initially treated for unstable angina. Following more investigation, echocardiography revealed a mild case of tricuspid regurgitation with an ejection fraction of 67%. The exercise stress test demonstrated a positive stress test with minimal effort. Coronary angiography results indicated positive vasospasm despite normal coronary arteries. The revised diagnosis was vasospastic angina.

A few months later, the patient reported left lower chest pain, claiming it worsened during deep inspiration and happened during rest. It was described as non-radiating and pin-pricking in nature and associated with tenderness on palpation over the fifth and sixth intercostal ribs. She had a normal ECG and troponin T. A costochondritis diagnosis was made after ruling out other potential explanations. Months later, the patient became aware of an odd lump above her left breast associated with weight loss, fatigue, and shortness of breath.

On examination, faint bilateral areolar scars were seen, but the breasts looked symmetrical. Upon contraction of the pectoralis muscle, left breast irregularities can be felt. The bilateral breasts were soft to firm, with an irregular, compressible lesion at seven o’clock, non-tender, and no lump on the right breast. There were no axillary or supraclavicular lymph nodes palpable. Bilaterally, the breast does not appear to be ptotic.

**Figure 1:**
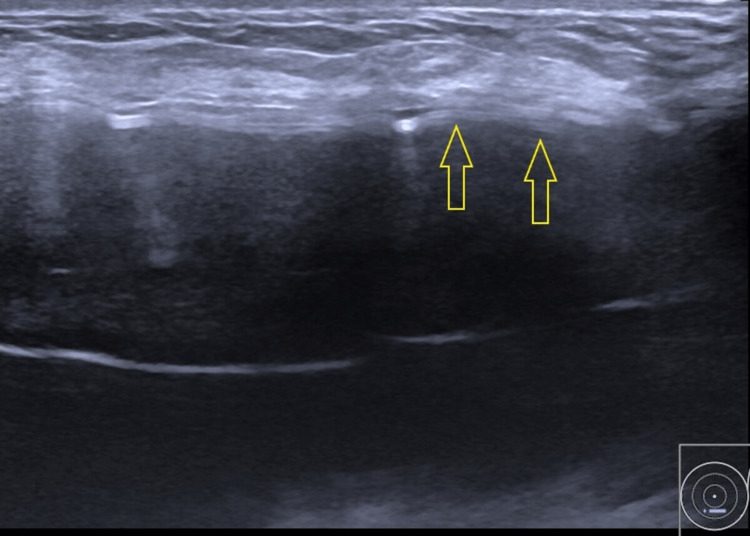
Left breast ultrasound showing a stepladder sign (yellow arrows), indicating an intracapsular implant rupture.

The stepladder sign was seen on the left breast ultrasound, as shown in Figure 1. This sign is suggestive of an intracapsular implant rupture. Unfortunately, the usefulness of a mammogram for this patient was limited due to the technician's limited experience with breast implants at our facility. Other than that, the right breast seems to be normal. The patient's claustrophobia precluded the use of an MRI. 

**Figure 2:**
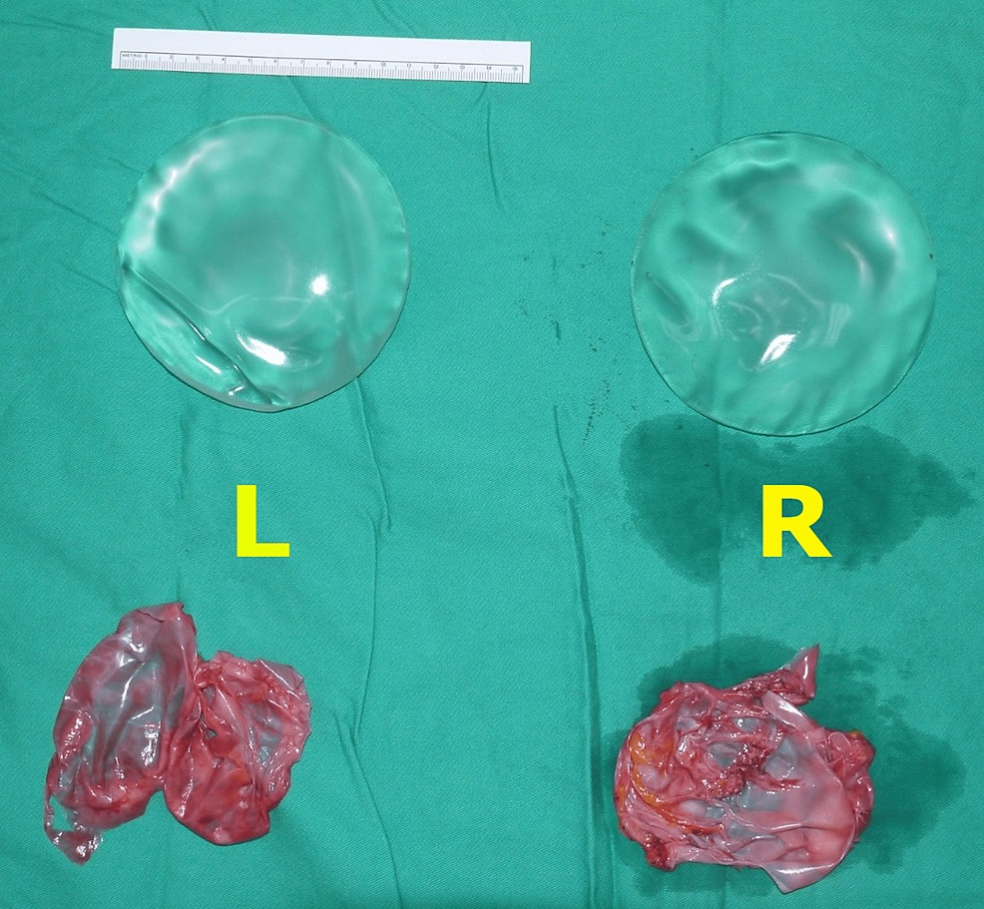
Left (L) and right (R) breast implants and their capsules post-explantation and capsulectomy.

The patient underwent bilateral breast implant removal and complete capsulectomy (Figure 2) with bilateral mastopexy without breast augmentation as per the patient's request. Additional laboratory tests were conducted as the patient displayed indications and symptoms of breast implant-associated anaplastic large cell lymphoma (BIA-ALCL). The tests include left breast biopsies, which were sent for histopathology and immunochemistry. Flow cytometry of T-cell clones is not done at our facility.

Post-operatively, the surgery was uneventful, and the patient was discharged after a few days. The incision was entirely healed on follow-up, the histopathological report of the samples denied any evidence of atypical large anaplastic cells, and the attached breast tissues were unremarkable. There were small reactive lymphocytes composed of a mixed population of both B (CD20+) and T (CD3+) cells. However, the CD30 was negative. The histopathological study of the right breast implant revealed ductal hyperplasia and fibrocystic change. Mammography post-operatively reported bilateral benign breast calcifications with post-operative changes. Regarding the presenting symptoms, they all resolved post-operatively.

## Discussion

The presence of chest or precordial pain or discomfort is the main characteristic symptom of Acute Coronary Syndrome (ACS). The chest pain or discomfort is frequently described as feeling heavy, tight, squeezing, or like an elephant sitting on the sufferer's chest. The symptom may spread to the jaw, neck, shoulders, right or left arm, back, and the area around the epigastric region of the upper torso. Shortness of breath, nausea, vomiting, diaphoresis, or lightheadedness are just a few of the symptoms that may accompany it frequently. Even though the symptoms typically manifest while at rest, exercise may worsen them. The strength, seriousness, length, and potential crescendo character of ACS symptoms can all differ. The symptoms of ACS might develop suddenly or slowly over several days, weeks, or months [5]. Atypical pain was described as "epigastric or back pain or pain that was burning, stabbing, characteristic of indigestion, or other" [6].

It is crucial to rule out cardiac reasons before looking into potential non-cardiac sources when patients seek therapy for chest pain. Depending on the clinical presentation and cardiac risk factors, the initial cardiac workup should start with a history and physical examination and be followed by diagnostic tests that can differ from patient to patient [7]. Acute coronary events are a major consideration for people with chest pain since they are the most common cause of mortality in women, with over half of all fatalities in women over 50 being related to cardiovascular disease [8]. However, if the findings of a stress test and other cardiac tests are improbable, as they were in this patient's case, breast implant issues should be taken into consideration as part of the differential diagnosis of chest pain in a middle-aged woman with implants. As with this patient, persistent chest pain develops slowly after breast augmentation. Her complaints remained unresolved despite multiple doctor visits. The patient could not recall the texture, shape, and content of the breast implant used.

When an implant is inserted, the body forms a fibrous capsule around the implant. A ruptured saline implant causes the innocuous extravasation of an isotonic saline solution that the body will reabsorb over time. However, silicone implant ruptures can result in additional complications. For example, in an extracapsular rupture, silicone gel extravasates into the adjacent tissues, frequently in an infiltrative pattern that can result in a significant local tissue reaction and scar formation [9]. In addition, there is a difference between older silicone and newer, more cohesive silicone implants. Without certain exceptional circumstances, cohesive silicone will not spread to adjacent tissue; however, silicone present in older generations of implants is susceptible to widespread dissemination. In this case, the patient underwent breast augmentation with implants 10 years ago, which were fifth-generation breast implants whose shell and gel viscosity were redesigned according to strict criteria by the FDA [10]. Silicone implant rupture is difficult to detect on physical examination, as most cases are intracapsular; imaging is often required for further evaluation. Extracapsular silicone implant ruptures may produce a palpable mass or breast irregularity as inflammation and granulomatous tissue develop [9].

The next important step is to accurately identify a non-cardiac cause of pain as soon as possible, ideally within a year of the onset of symptoms. Establishing non-cardiac chest pain could be done under four categories, which involve assessment of cardiac risk factors, quality of chest pain, risk factors for psychological and other disorders, and the patient's concerns and worries [11]. Although there was a strong suspicion of heart illness given our patient's medical history and symptoms, her angina-like symptoms were finally determined to be caused by her left breast implant intracapsular rupture.

Lee et al. found that 11 patients with silicone breast implants developed episodes of severe chest discomfort that resembled heart attacks six weeks to seven years following breast implantation. The chest pain, unrelated to physical effort, might last between 15 minutes and four days. It was described as "pressing" or "stabbing," radiating to the shoulders, left arm, and jaw. The findings of all ten cardiac tests, which were all performed, were all normal. Five patients experienced at least one ruptured implant, and all had their implants removed. They concluded that silicone breast implants could result in unusual chest pain, most likely due to localized inflammatory responses and neuroma formation [7].

Most breast implant rupture patients have "silent" ruptures, meaning they do not exhibit clinically relevant indications or symptoms [12]. Patients' symptoms, such as changes to their breasts' size, shape, or firmness, capsular contracture, palpable lumps, or breast pain, primarily prompt an evaluation for implant rupture. The patient displayed these symptoms, and early intervention prevented future complications.

It is generally known that silicone can cause several unfavorable reactions in tissues. In two experiments on the bioreactivity of silicone conducted in 1987, Kossovsky et al. showed that silicone could physically cause a chronic inflammatory process in human tissue that is immunologically driven, resulting in the contraction and encapsulation of fibrous tissue [13,14]. Even though many women experience various complications with breast implants, few previous studies have shown a causality between silicone implants and cardiovascular disease. However, silicone implants are still mostly considered safe to use. Moreover, case-control research has indicated that breast implants are linked to an age-related increased risk of developing BIA-ALCL, and several recent articles report issues related to silicone breast implants [15]. In 2011, the FDA issued its initial statement delineating the potential link between breast implants and lymphoma. Epidemiologic risk estimation indicated that the risk of developing BIA-ALCL from all breast implants was as low as 1 in 500,000 [16]. Fortunately, the pathology results were negative in this case despite the patient exhibiting symptoms associated with BIA-ALCL.

Patients with silent rupture should be given the choice of surgery or observation, as has been inadvertently documented. Many choose to observe with interval studies. Patients who have additional clinical issues that surgery may be able to address are more motivated. Removing the implants, typically involving a complete capsulectomy, is the advised surgical course of action for identified or symptomatic ruptured silicone breast implants. If the rupture is intracapsular, meaning that the surrounding fibrous capsule confines the free silicone, explantation entails the removal of the implant and any free silicone, capsulectomy, and replacement of the implant if the patient so chooses. A similar treatment is used for the extracapsular rupture with free silicone in the breast tissue, with the addition of resecting any visible or palpable granulomas in the breast parenchyma [17]. A 2015 analysis examined silicone's potential connection to the Autoimmune/inflammatory Syndrome Induced by Adjuvants (ASIA). They concluded that there is growing evidence of this connection and discussed silicone's capacity to directly mediate cellular immune responses, which can lead to inflammation and autoimmune disorders. They also proposed silicone as the "missing link" as an environmental trigger, suggesting that certain patients may have a genetic propensity to develop autoimmunity [18]. The overall risk of complications after breast implant surgery is 27.6%, with 23.1% of patients needing another deflation or capsular contracture procedure. The early complications are typically defined as those that occur within one month of surgery and include hematoma, seroma, wound dehiscence, infections, and Mondor's disease.

Our patient developed breast discomfort, an abnormal lump, and capsular contracture, particularly in the left breast. These are late complications associated with breast implants. Additionally, the patient's complaints of weight loss, exhaustion, and breathlessness raised the possibility of BIA-ALCL, a rare disease linked to breast implants [19]. The National Comprehensive Cancer Network (NCCN) has standardized recommendations for treating BIA-ALCL [19]. In most cases, complete surgical resection provides definitive treatment and cure. However, a partial resection or poor local surgical control may demand additional treatments for the patient in the interim (i.e., chemotherapy or radiation therapy) [20].

Multiple diagnostic modalities may be used to evaluate implant rupture, including MRI, ultrasound, mammography, and CT scanning. Currently, the FDA advises women with silicone breast implants to have screenings for implant rupture three years after implantation and every two years after that [9].

## Conclusions

A woman who had chest pain brought on by a ruptured breast implant but who also displayed various false symptoms of coronary artery disease. The negative coronary artery disease test findings motivated us to examine the breast implant integrity, which led to the proper diagnosis and recommended intervention. Any patient who exhibits chest pain or other signs of coronary artery disease and has breast implants should consider breast implant late complications when making a differential diagnosis. However, since coronary artery disease is much more prevalent in middle-aged women than complications from breast implants, it should continue to be the top priority. Until proven otherwise, any woman with breast implants experiencing atypical chest pain should undergo a breast ultrasound prior to a comprehensive cardiac evaluation.
